# A fingerprints based molecular property prediction method using the BERT model

**DOI:** 10.1186/s13321-022-00650-3

**Published:** 2022-10-21

**Authors:** Naifeng Wen, Guanqun Liu, Jie Zhang, Rubo Zhang, Yating Fu, Xu Han

**Affiliations:** 1 School of Mechanical and Electronic Engineering, Dalian Minzu University, Dalian, China; 2 Beijing Huawei Digital Technologies Co., Ltd, Beijing, China

**Keywords:** Molecular property prediction, Molecular representation, Pre-training language model, Deep neural network, Quantitative structure-activity relationships

## Abstract

Molecular property prediction (MPP) is vital in drug discovery and drug reposition. Deep learning-based MPP models capture molecular property-related features from various molecule representations. In this paper, we propose a molecule sequence embedding and prediction model facing with MPP task. We pre-trained a bi-directional encoder representations from Transformers (BERT) encoder to obtain the semantic representation of compound fingerprints, called Fingerprints-BERT (FP-BERT), in a self-supervised learning manner. Then, the encoded molecular representation by the FP-BERT is input to the convolutional neural network (CNN) to extract higher-level abstract features, and the predicted properties of the molecule are finally obtained through fully connected layer for distinct classification or regression MPP tasks. Comparison with the baselines shows that the proposed model achieves high prediction performance on all of the classification tasks and regression tasks.

## Introduction

Molecular property prediction (MPP) is an important issue in drug design and substance discovery. It is conducive to improving chemical design, reducing research and development costs and accelerating the process of drug discovery. According to the different predicted properties, the molecular property prediction problem can be divided into classification tasks (such as toxicity) and regression tasks (such as atomization energy). Traditional methods based on density functional theory have explicit physical images but are time consuming when processing large numbers of molecules. In recent years, the prediction of compound properties based on machine learning has attracted extensive attention from researchers, among which quantitative structure-activity relationships (QSAR) are one of the commonly used methods. The main idea of QSAR is that the structure of a molecule determines its properties; that is, the biological activity of a compound can be predicted by its molecular structure. Another major application of QSAR is virtual screening in drug discovery, which reduces the number of candidate compounds that need to be experimentally tested, thus reducing development costs and speeding up the drug discovery process.

Traditional QSAR methods use classical machine learning methods such as support vector machines (SVM) and random forests. However, in a 2012 Kaggle competition (Merck Molecular Activity Challenge), the champion team used the deep learning method to increase the accuracy rate by 15% compared with the traditional method [1]. Ma et al. [2] compared the performance of a deep learning model with random forest on a set of QSAR datasets, including the Kaggle dataset, and found that the performance of the deep learning method was better in most cases. Xu et al. [3] applied a multitask neural network and discussed the reasons for performance differences caused by multitasks. The successful application of these deep learning techniques greatly improved the accuracy of the QSAR method, which led to more extensive research.

Deep learning techniques have been widely used in molecular property prediction. Yang et al. [4] compared two different models for predicting molecular properties, one using fixed molecular fingerprints/molecular descriptors and the other using graph convolutional neural networks to learn molecule representations. Liu et al. [5] proposed a multilevel graph convolutional neural network (MGCN), which predicted molecular properties based on density functional theory. Wang et al. [6] proposed a molecular embedding layer based on graph convolution, but it also retained molecular fingerprints to enhance generalization performance. Jeon and Kim [7] proposed FP2VEC, a molecular featurizer based on molecular fingerprints, which represented each compound as a group of trainable vectors and built a QSAR model to verify the ability of FP2VEC to extract molecular features. The above molecular embedding method achieved good performance, but it must be carried out under supervised conditions.

Natural language processing (NLP) takes human language as the research object, and the techniques used in this field can also be applied to biological data. Some models explicitly refer to the encoders in NLP, treating molecules as sentences and atoms or substructures as words, thus achieving various embeddings of molecules. These encoders capture the generalizable features of molecular via self-supervised learning and subsequently transfer the pre-trained embedding model to downstream tasks. For example, the FP2VEC method treated the substructures obtained by the molecular fingerprint algorithm as words and performed word embedding [7]. The Mol2Vec method used the word2vec model for the substructures [8].

Recently, many remarkable pre-trained models for learning the representations of chemical molecules have been proposed based on the Transformer model, specifically the bi-directional encoder representations from Transformers (BERT) model [9–15]. Compared to word2vec, BERT consists of multiple Transformer encoders that can capture contextual information simultaneously to learn the word vector that integrates contextual information [16, 17].

A proper molecular representation method is essential for molecular property prediction. Most of the Transformer- and BERT-based models take as input of the common simplified molecular-input line-entry system (SMILES) strings. They often adopt the atom-level tokenization that usually ignores substructure or branch information of molecules to some extent [18]. And that tokenization may also result in simplicity of the training tasks. Besides, the SMILES may cause a large number of ‘synonyms’ in the vocabulary [13].

Compared with atom-level representations, substring-level representation provides some substructure information or fragments of the molecule in detail [18]. The vocabulary for the substring-level representation is physicochemically meaningful due to the fact that several atoms can form small atomic groups, which can further form larger atomic groups, and then these larger groups constitute molecules [18].

Thus, we built the substring-level vocabulary by using the extended-connectivity fingerprints (ECFP) generation algorithm [8, 19] on a big corpus. Then the molecular sentences can be captured as the model input.

Meanwhile, various task-specific pre-training strategies were explored in [9, 11–13, 15]. The MolBERT devised the pre-training strategies of SMILES equivalence and predicting the normalized set of descriptors for each molecule [9]. The X-MOL designed a generative model by generating a valid and equivalent SMILES representation of the same molecule [11]. However, the strategy is not from a language-modelling perspective. Thus the Chemformer explored the pre-training tasks of short sequence masking and SMILES similarity [12]. The K-BERT employed the atom feature prediction, molecular feature prediction and contrastive learning pre-training tasks [13].

Motivated by the successful applications of the BERT in molecular encoding [9, 20], we propose a molecule property prediction framework composed of a pre-trained BERT encoder called Fingerprints-BERT (FP-BERT) to obtain the semantic representation of a molecule, by self-supervised learning using a corpus containing millions of molecule sentences. Then, the encoded molecular representation by the FP-BERT is input to the convolutional neural network (CNN) to extract higher-level abstract features, and the predicted properties of the molecule are finally obtained through fully connected layer for distinct classification or regression MPP tasks, such as the Absorption, Distribution, Metabolism, Excretion and Toxicity(ADME/T) prediction.

Different from the mainstream, we employed the molecular sentences to pre-train the BERT encoder, and we explored the substructure masking pre-training task. The novelties of this paper are summarized below:

1. We take molecular sentence as the model input to pre-train the BERT encoder by predicting the masked substructural features of a molecule;

2. We built the physicochemically meaningful vocabulary for substructures, to leverage the atomic neighbor information in molecular representations.

## Methods

**Fig. 1:**
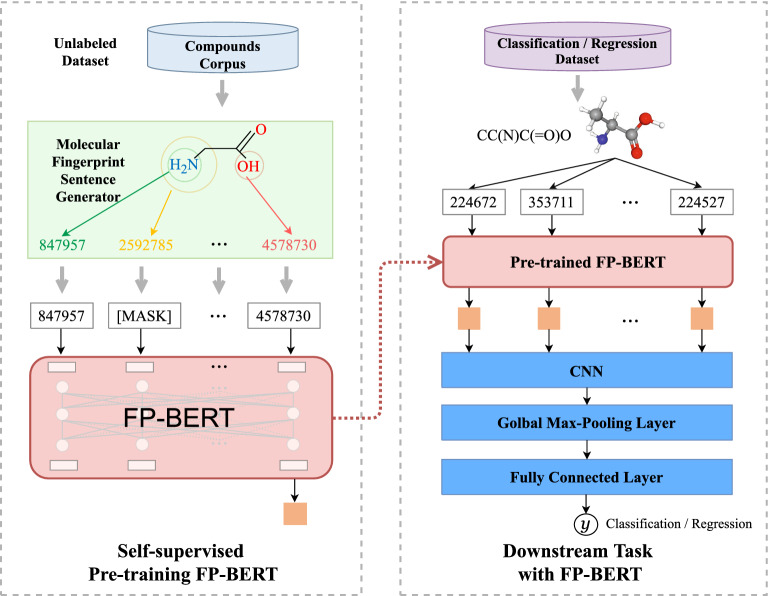
The architecture of FP-BERT based MPP model

The framework of FP-BERT based MPP framework is shown in Fig. 1. The proposed MPP method in this paper consists of two parts: the pre-trained FP-BERT model on the left; and the neural network for the downstream prediction tasks on the right. To pre-train the FP-BERT, a large number of unlabeled compound molecules in the form of SMILES are converted into an ECFP [19] of radius 1 (as shown in Fig. 1 on the upper left) using the RDKit [21], and then a list of substructure identifiers and molecular sentences are obtained by molecular fingerprint sentence generator. Herein, a corpus containing 2 million molecular sentences is built up and fed into the BERT model in a self-supervised learning manner to obtain a pre-trained FP-BERT encoder. In the downstream prediction model, a neural network consists of the pre-trained FP-BERT as the input encoder, the CNN layer, a global max-pooling layer and fully connected layer. The network is trained in a supervised manner with the FP-BERT fixed according to various downstream molecular property prediction tasks.

### Molecular fingerprint encoding based on a language model

Inspired by self-supervised learning in NLP, we regard the compound substructures derived from the Morgan algorithm as words and the compounds as sentences to construct a corpus of compound molecules. Then, this corpus is used to pre-train the BERT model. The pre-trained BERT model can generate a high-dimensional embedding representation of the substructure for any compound. Thus, any compound represented by substructures is converted to a molecular representation in vector form, and downstream tasks such as molecular property prediction can be further completed.

####  Generation of molecular sentence

In NLP, each sentence consists of a sequence of words. In chemistry, each molecule consists of a set of molecular substructures. Thus, a compound can be understood as a sentence, each substructure as a word, and the encoding of the entire compound can be obtained by representing each substructure as a vector. To enumerate the substructures in compounds and encode them, we first use the Morgan algorithm [22] to generate ECFP fingerprints and extract the substructures (i.e., “words”) existing in the compounds and then generate the corresponding molecular sentence for each molecule. We randomly select compounds from the E15 [23] dataset. The E15 compound dataset is the diverse real drug-like subset of the ENA dataset provided by the Enamine Corporation, and it contains 15.5M molecules and their SMILES representations.

The initial SMILES representation of a compound is an ASCII string *S*= “$s_1 s_2 s_3 \cdots s_n$ ”, where $s_i$ ($i \in \{ 1,2,\cdots ,n\}$) can be Arabic numerals, English letters and special symbols. These characteristics in the SMILES string are used to represent atoms or chemical bonds in the compound. For example, a SMILES representation of 1-nitropropane is CCCN(=O)=O, where ‘(’ and ‘)’ denote the beginning and the end of the branch, ‘=’ represents a double bond, and ‘C’, ‘N’ and ‘O’ denote the carbon atom, nitrogen atom and oxygen atom, respectively.

**Fig. 2:**
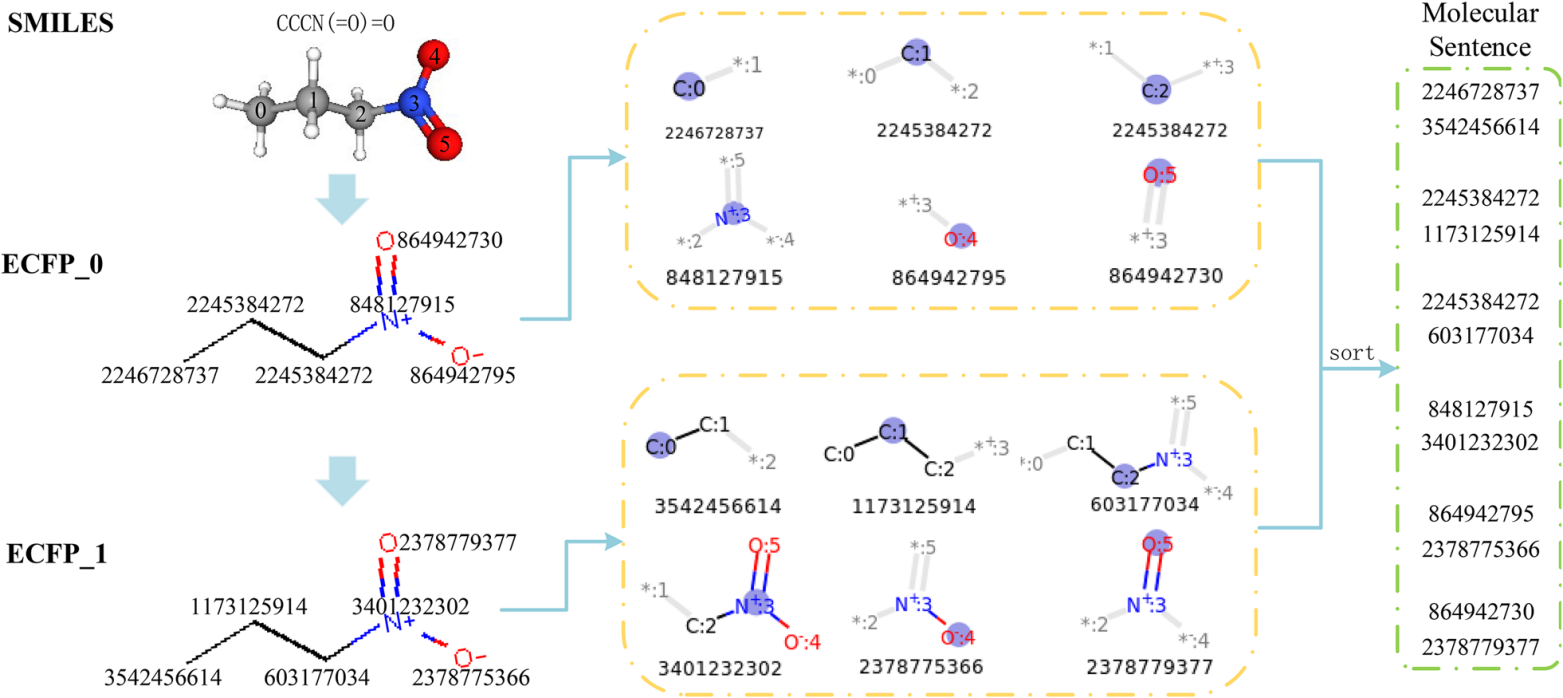
The generation of molecular sentences

In this paper, the algorithm is used to generate the corresponding ECFP fingerprint from the SMILES representation to construct the molecular sentence [19, 22]. The main idea is to take each atom $s_i$ in the sequence of SMILES as the center and find substructural fragments $s_{i0}$ and $s_{i1}$ with radius 0 and radius 1 in the molecular structure. $s_{i0}$ contains the information of the atom, while $s_{i1}$ contains the neighboring node information. Then, the two substructures $s_{i0}$ and $s_{i1}$ generated by atom $s_i$ are mapped into the corresponding substructure identifiers $a_i^0$ and $a_i^1$, respectively. In addition, all substructure identifiers are sorted according to the order of each atom in the SMILES string and the radius of the substructure to obtain an atom identifier sequence *L*=[$a_1^0,a_1^1,a_2^0,a_2^1, \cdots , a_p^0, a_p^1$], where $a_i^0$, $a_i^1$ ($i\in$ 1, 2, $\cdots$, *p*) are 4-byte integers and *p* represents the number of atoms in the SMILES sequence. *L* is the molecular sentence of the molecule, and its generation process is shown in Fig. 2. The specific steps of the above generation algorithm are shown below.

The molecular sentence generation process has three sequential stages.

1. Each atom (except the hydrogen atoms and bonds to hydrogen atoms) is initially assigned a fixed length integer identifier $a_i^0$ that is hashed from the properties of the atom *i* and its attached bonds. An integer can be regarded as an indexes of a virtual bit string, and a bit of the virtual string indicates the existence(s) of a substructure or substructures. The hash function is used to map atom properties of arbitrary size to fixed-size integer identifiers to improve the storage efficiency. The property set consists of the properties, such as atomic number, the number of adjacent heavy atoms (non hydrogen atoms) of the central atom, the number of adjacent hydrogen atoms of central atom, formal charge, and an additional attribute: whether the atom is a part of the ring. These integer identifiers are collected to form the initial fingerprint set $L^0$ = $\{a_1^0,a_2^0, \cdots , a_p^0 \}$;

2. The integer identifier set is updated iteratively. At the first iteration, substructures centering at initial atoms with radius 1 are matched. Then the integer identifier $a_j^1$ for the *j*th substructure is captured by hashing, and all the newly generated identifiers are added to the fingerprint set. The identifiers for iteration 1 contain information about each atom’s immediate neighbors. At the *t*th iteration, the identifier for the *k*th substructure with radius *t* is updated to $a_k^t$, and the generated identifier for each substructure is added to the fingerprint set of the last iteration to form a new fingerprint set $L^t=\{a_1^0, a_2^0, \cdots , a_p^0, a_1^1, a_2^1, \cdots , a_p^1, \cdots , a_1^t, a_2^t, \cdots , a_p^t \}$;

3. The updating process iterates until the substructure radius reaches a specific threshold, then duplicate or equivalent identifiers are removed. Finally, according to the atomic order in the canonical SMILES and the radiuses of substructures, sort all the identifiers in the fingerprint set to obtain the molecular sentence *L*.

#### Pre-training description of BERT

The self-supervised pre-training of this study is conducted using respective corpuses contains millions of unlabeled compounds which are all processed according to the molecular sentence generation method introduced in 2.1.1, resulting in 3352 atom identifiers (words). The dictionary used in this paper has a total of 3357 words. In addition to these atom identifiers, it also contains five special words [PAD], [UNK], [CLS], [SEP], and [MASK]. Each molecule in the corpus is a sentence composed of substructure identifiers. These molecular sentences are used as the word embedding vectors in the input sequence of the BERT model, and the segmentation embedding vectors and the position embedding vectors are concatenated to the input sequence. These three embedding vectors are sent to the Transformer encoder to learn the representation of the compound. The most important module in BERT is the self-attention mechanism. The self-attention mechanism adjusts the weight of each word in the input sequence to obtain a global representation vector containing the context.

**Fig. 3:**
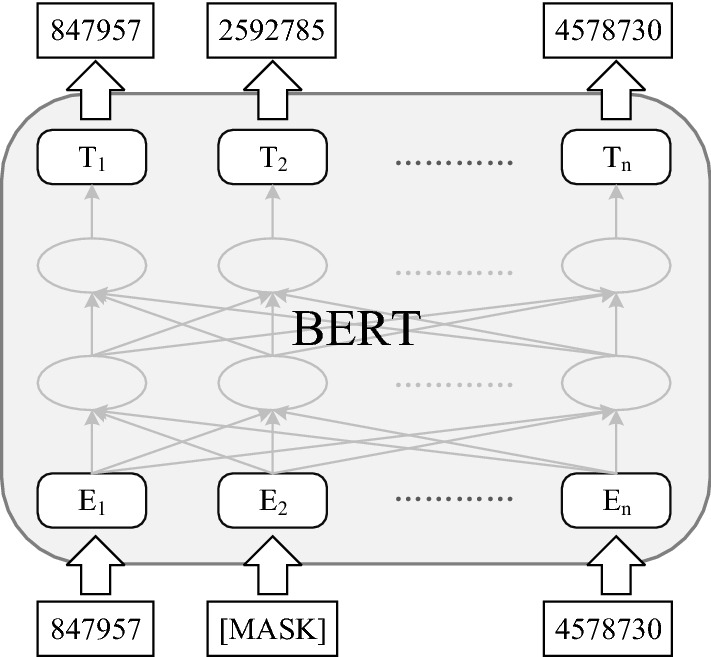
Pre-training procedures for BERT

In this paper, the task of pre-training FP-BERT is a masked language modeling (MaskedLM), which randomly masks a portion of the words in the input sentence and attempts to predict those masked words. As shown in Fig. 3, the MaskedLM task randomly covers up to 15% of words in each sentence composed of substructures in the training corpus and attempts to predict those words that are covered. For those covered words, the following three strategies are adopted:

1. Replace the masked word with [MASK] with 80% probability;

2. Replace the masked word with a random word with a probability of 10%;

3. Stay the same with 10% probability.

The pre-training task MaskedLM can make the FP-BERT model more dependent on the contextual information to predict the masked words, which gives the model a certain degree of error correction capability [24].

After the pre-training process is completed, taking the molecular sentence of any compound as input, the FP-BERT model generates the encoded representation of the molecule. The output of the model is a list of the state vector $T_i \in {\mathbb {R}}^H$ corresponding to each compound substructure, where *H* represents the hidden size and $i \in \{1, 2, \cdots , n \}$. All substructure vectors form the encoded representation *T*=[$T_1,T_2,\cdots ,T_n$] of the compound.

### Molecular property prediction model

After obtaining the appropriate molecular representation, a prediction model can be constructed to predict the molecular properties. CNNs can capture the local features of grid-like data and have been successfully applied in the field of image processing and natural language processing. In this paper, we build a molecular property prediction model based on the CNN framework. This CNN-based prediction model is mainly composed of a one-dimensional convolutional layer, a global max-pooling layer, and a fully connected layer.

First, we use a 1D convolutional neural network to perform feature extraction on the compound representation vector obtained by the BERT model according to

1 $$\begin{aligned}&o_{conv} = Conv1d(x_{in} \circledast w_{conv}) \end{aligned}$$

where $x_{in} \in {\mathbb {R}}^{n \times 256}$ represents the learned molecular representation, *n* represents the number of tokens in the input sequence, $\circledast$ represents the convolution operation, $w_{conv}$ represents the parameters to be learned, and $o_{conv}$ represents the output after passing through the 1D convolutional network. In addition, after the convolution layer, we use ReLU as the activation function.

After feature extraction by CNN, max feature $o_{max}$ can be selected from $o_{conv}$ by the max-pooling operation to achieve dimensionality reduction and parameter sharing.

Finally, the fully connected network outputs the prediction results of the molecular properties of the compound according to

2 $$\begin{aligned}&y = \mathbf {f}(\mathbf {o}_{\textbf {max}} \cdot \mathbf {w}_{\mathbf {fc}} + {\mathbf {b}}_{\mathbf {fc}}) \end{aligned}$$

where $w_{fc}$ represents the parameter matrix of the fully connected layer, $b_{fc}$ represents the deviation, *f* represents the ReLU activation function, and *y* represents the prediction results of the CNN model. In the classification task, *y* is a one-hot vector, which represents whether the molecule has the current property, and in the regression task, *y* is a real number, which represents the specific property value of the molecule.

For the classification task, we use the cross-entropy loss function to optimize our model according to

3 $$\begin{aligned} &Loss = -\frac{1}{n} \sum _{i=1}^{n} t_i \cdot \log y_i + (1-t_i) \cdot \log (1-y_i) \end{aligned} $$

where $t_i$ represents the true label of sample *i*, $y_i$ represents the probability that sample *i* is predicted to be a positive sample, and *n* represents the number of samples.

For the regression task, we use the mean squared error loss function to optimize our prediction model according to

4 $$\begin{aligned}&Loss = -\frac{1}{n} \sum _{i=1}^{n} (y_i - t_i)^2 \end{aligned} $$

where $t_i$ represents the real property value of sample *i*, $y_i$ represents the predicted property value of sample *i*, and *n* represents the number of samples.

### Performance evaluation method and hyperparameter setting

In this paper, the molecular property prediction model used ReLU as the activation function. For classification data sets, the prediction performance of the model was evaluated using ROC-AUC, which represents the area under the receiver operating characteristic curve. The calculation process of AUC is shown as follows,

5 $$\begin{aligned} &AUC = \frac{1}{M \times N} \left[ \sum _{i \in positive} rank_i - \frac{M \times (1+M)}{2} \right] \end{aligned}$$

where *M* and *N* represent the number of positive and negative samples, respectively, and $rank_i$ represents the ranking of the score of sample *i* among all *n* samples.

For regression datasets, the prediction performance of the model was evaluated using root mean squared error. The calculation process of *RMSE* is shown as follows:

6 $$\begin{aligned} &RMSE = \sqrt{\frac{1}{n}\sum _{i=1}^n (y_i - t_i)^2} \end{aligned} $$

where $t_i$ represents the real property value of sample *i*, $y_i$ represents the predicted property value of sample *i*, and *n* represents the number of samples.

The $R^2$ metric reflects the goodness of fit and it is calculated on the training set wherein *RSS* is the residual sum of squares and *TSS* is the total sum of squares, and ${\overline{y}}$ is the mean of the predicted values.

7 $$\begin{aligned}&R^2 = 1-RSS/TSS\\&RSS = \sum _{i=1}^n(y_i-t_i)^2\\&TSS = \sum _{i=1}^n ({\overline{y}} - t_i)^2 \end{aligned}$$

The $Q^2$ metric reflects the goodness of prediction and it is calculated on the test set wherein *PRESS* is the predictive residual error sum of squares.

8 $$\begin{aligned} &Q^2 = 1-PRESS/TSS\\&PRESS = \sum _{i=1}^{n} (y_i - t_i)^2 \end{aligned} $$

## Results and discussion

To evaluate the performance of the FP-BERT model, we conducted comparison experiments on five regression datasets and two classification datasets for molecular property prediction. On the HIV and the BBBP classification datasets, we compared the FP-BERT with the benchmark models: FP2VEC [7], MolBERT [9], FCNN [25] and Bypass [25]. On the regression datasets ESOL, Freesolv, and Lipophilicity, we compared FP-BERT with the benchmark models MolBERT, FP2VEC and FCNN. On regression datasets Malaria and CEP, we compared FP-BERT with the benchmark models FP2VEC and ECFP. In addition, to validate the influence of the corpus size in the pre-training of FP-BERT, we also provide the results of the FP-BERT pre-trained on 10 million compound molecular sentences.

### Datasets

In the experiment, we used the HIV dataset and the BBBP dataset for classification, and the ESOL dataset, the FreeSolv dataset, the Lipophilicity dataset, the Malaria dataset, and the CEP dataset for regression, to train and validate the proposed MPP model. These datasets are taken from the literatures [25] and [26]. In each dataset, the compound is represented as a SMILES string. The datasets are described as follows.

- HIV dataset [27]: The HIV dataset is an experimental measurement of the ability to inhibit HIV replication. The HIV dataset contains 41,127 compounds and their ability of inhibition with binary labels.
- BBBP dataset [28]: The BBBP dataset is blood-brain barrier penetration with binary labels. The dataset has a total of 2050 compounds.
- ESOL dataset [29]: The ESOL dataset includes measurements of the water solubility of small compounds. Water solubility is represented as a measured log solubility in moles per liter. The ESOL dataset includes 1128 compounds and their water solubility.
- FreeSolv dataset [30]: The FreeSolv dataset contains the hydrogen-free energy of small compounds in a water environment measured by experiment and computer simulation. The dataset contains 642 molecules and their hydrogen-free energy.
- Lipophilicity dataset: The Lipophilicity dataset contains an octanol/water distribution coefficient at pH 7.4 measured experimentally. The dataset has 4200 compounds and their corresponding values.
- Malaria dataset [31]: The Malaria dataset includes the experimentally measured half-maximal effective concentration (EC50) values of a sulfide-resistant strain of Plasmodium falciparum, which is the source of malaria. The Malaria dataset has 9,998 compounds and their EC50 values.
- CEP dataset [32]: The CEP (Clean Energy Project) dataset includes the candidate molecules that are suitable for solar cell materials. The CEP dataset has 29,978 compounds and corresponding CEP values.

All datasets were divided into the training set, validation set and test set at a ratio of 8:1:1. The regression datasets used the random splitting method, while the classification dataset used the scaffold splitting method. The scaffold splitting method [33–35] splits the samples based on their two-dimensional structural frameworks, and it attempts to divide structurally different molecules into different subsets, then the structural differences of the compounds among the training, validation and test sets increase. Thus, the splitting offers a more difficult evaluation setting than the random splitting. And the scaffold splitting method can be used to testify the generalization of the model.

### Experiment setting

In the molecular representation learning process of the FP-BERT model, the embedding dimensionality of each substructure is 256. In the training process of the neural network model, the hyperparameters include the learning rate, the length of the convolution kernel, the number of convolution kernels, and the number of neurons in the fully connected layer. In the classification task, the learning rate is 0.001, the length of the convolution kernel is 5, the number of convolution kernels is 512, and the number of neurons in the fully connected layer is 256. In the regression task, the learning rate is 0.001, the length of the convolution kernel is 1, the number of convolution kernels is 2048, and the number of neurons in the fully connected layer is 256.

To avoid overfitting, we pre-trained the FP-BERT up to 40 epochs on all compound data sets with the early stopping scheme. In the experimental setup, we let the training of the models proceed until the accuracy parameter on a validation data set shows no sign of improvement for a given number of epochs, and then revert back to the best model found during the training.

### Experiments on classification tasks

For the classification task, we compare our FP-BERT based MPP models with the baselines, including the FP2VEC model [7], MolBERT model [9], FCNN model [25] and Bypass model [25]. And the featurizer FP-BERT are pre-trained by 2 million and 10 million compounds, respectively. Then, our MPP model is trained by the labeled data in the HIV or BBBP datasets to conduct the downstream task-specified classification. To make a fair comparison with the benchmark models, the datasets are prepared in the same way. To evaluate the accuracy of the prediction model, we use the average ROC-AUC of five independent experiments on the test set as the experimental result of the classification task. In addition, we use the standard deviation to measure the stability of the model. The experimental results of the classification task are shown in Table 1. We also ran the FP2VEC model and recorded the ROC-AUC value and the standard deviation. The experimental results of the MolBERT, FCNN and Bypass model are taken from the literatures [9, 25].

**Table 1:** The ROC-AUC scores on the test datasets

Model	Featurizer	HIV	BBBP
FP2VEC	FP2VEC	0.757 ± 0.006	0.713 ± 0.006
FCNN	ECFP	0.698 ± 0.037	0.688 ± 0.005
Bypass	ECFP	0.693 ± 0.026	0.702 ± 0.006
Ours-1	FP-BERT (2 M)	0.765 ± 0.006	0.696 ± 0.004
Ours-2	FP-BERT (10 M)	0.776 ± 0.005	0.714 ± 0.008
MolBERT	MolBERT	0.747 ± 0.000	0.750 ± 0.000

It can be observed that our models and the MolBERT achieve highest prediction performance in the classification task: the FP-BERT model (Ours-2) pre-trained on 10 million compounds captures the best result on HIV while the MolBERT achieves the best result on the BBBP dataset. Different from our models pre-trained only by the canonical masked language modeling (MaskedLM) task proposed by BERT, the MolBERT was pre-trained on two additional tasks: the SMILES-EQ and the PHYSCHEMPRED tasks. In the SMILES-EQ task, the MolBERT was trained to predict whether the two inputs represent the same molecule. In the PHYSCHEMPRED task, the MolBERT attempts to predict the normalized set of descriptors for each molecule. The task combination makes the encoder pre-trained more sufficiently and may lead to better feature representation ability. In addition, the PHYSCHEMPRED pre-training task is close to the down-stream QSAR task, possibly boosting the model predictive performance.

**Fig. 4:**
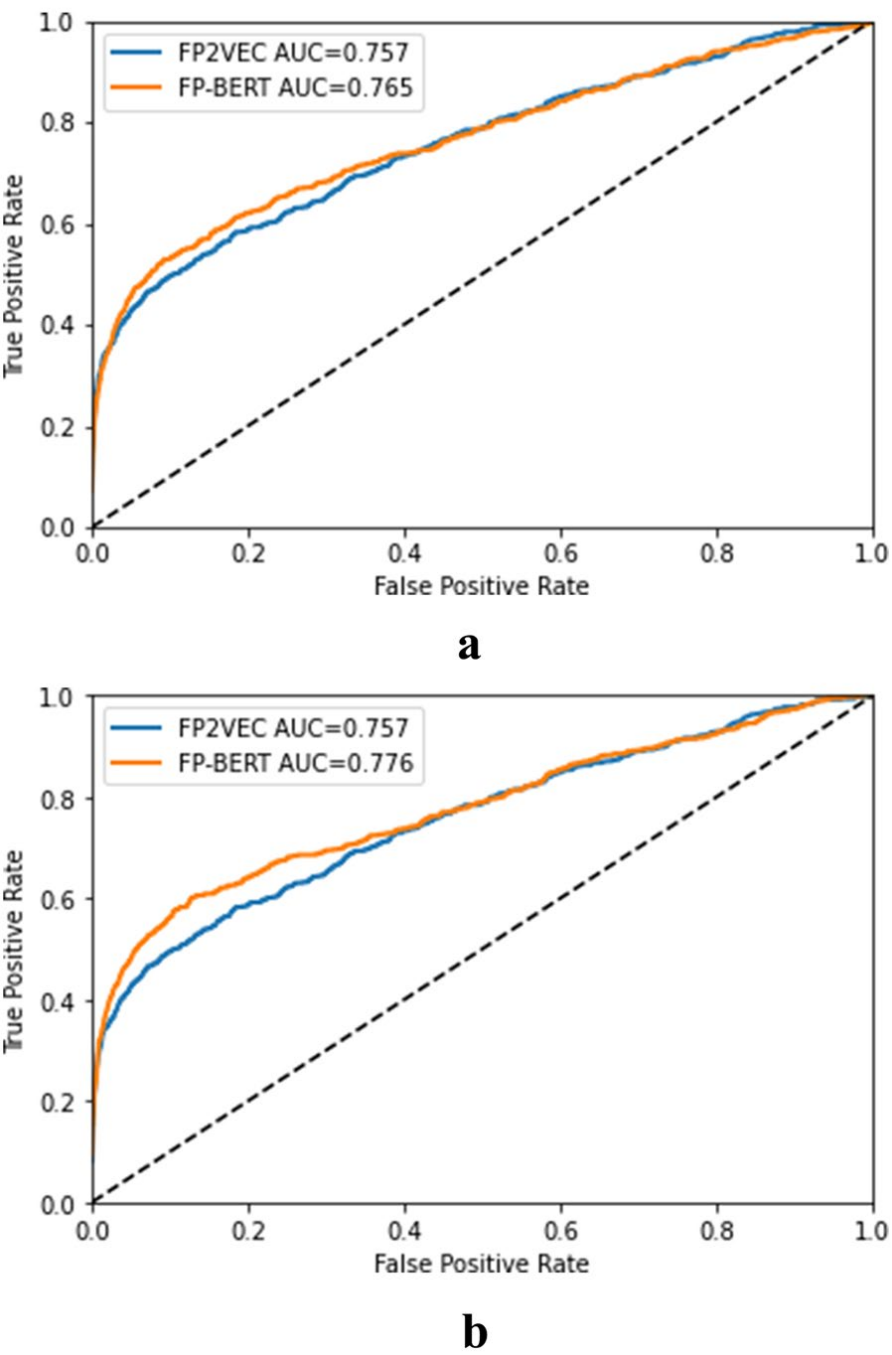
ROC curve on the HIV dataset

Compared to the MolBERT, the Ours-2 method pre-trained only on the MaskedLM task has reached the superior results to all the benchmarks on the HIV dataset and second best result on the BBBP dataset, meanwhile, the results of the Ours-1 model pre-trained on 2 million compounds are also competitive. The observations possibly indicate the effectiveness of our physicochemically meaningful vocabulary and taking molecular sentence as model input. The similar results can be found from the Fig. 4 that the ROC curves of our FP-BERT based MPP model almost cover the ROC curve of the FP2VEC model.

**Fig. 5:**
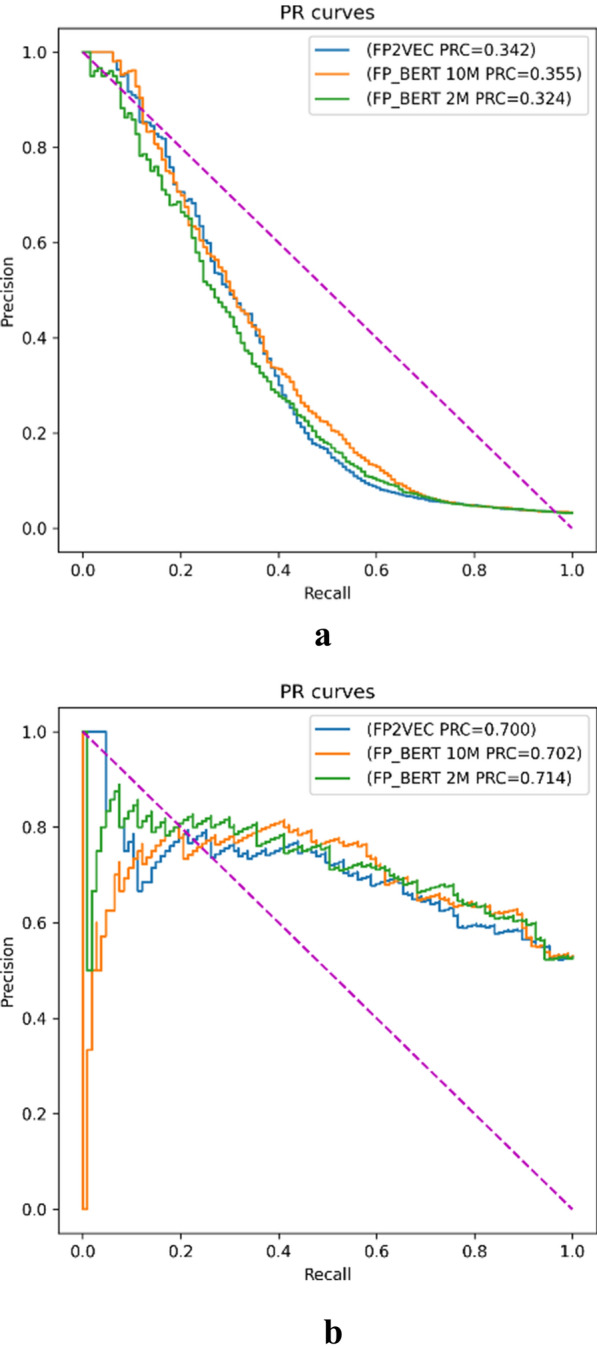
Comparative PR curves of our methods versus the FP2VEC method

Figure 5 shows comparative PR curves of our methods versus the FP2VEC method. On the HIV dataset, the Ours-2 model reaches the highest PR metric and the best PR curve, demonstrating the superior predictive performance. On the BBBP dataset, the Ours-1 model achieves the best PR value. The above observations can indicate the high performance of our FP-BERT featurizer.

In addition, the Ours-2 model obtains better results on both the HIV and BBBP datasets than the Ours-1. The observation may indicate that the BERT-style molecular representation model improves with bigger dataset in a range. But the prediction model performance depends on multiple factors, that may be related to the pre-training dataset size, the specific downstream dataset and task.

**Fig. 6:**
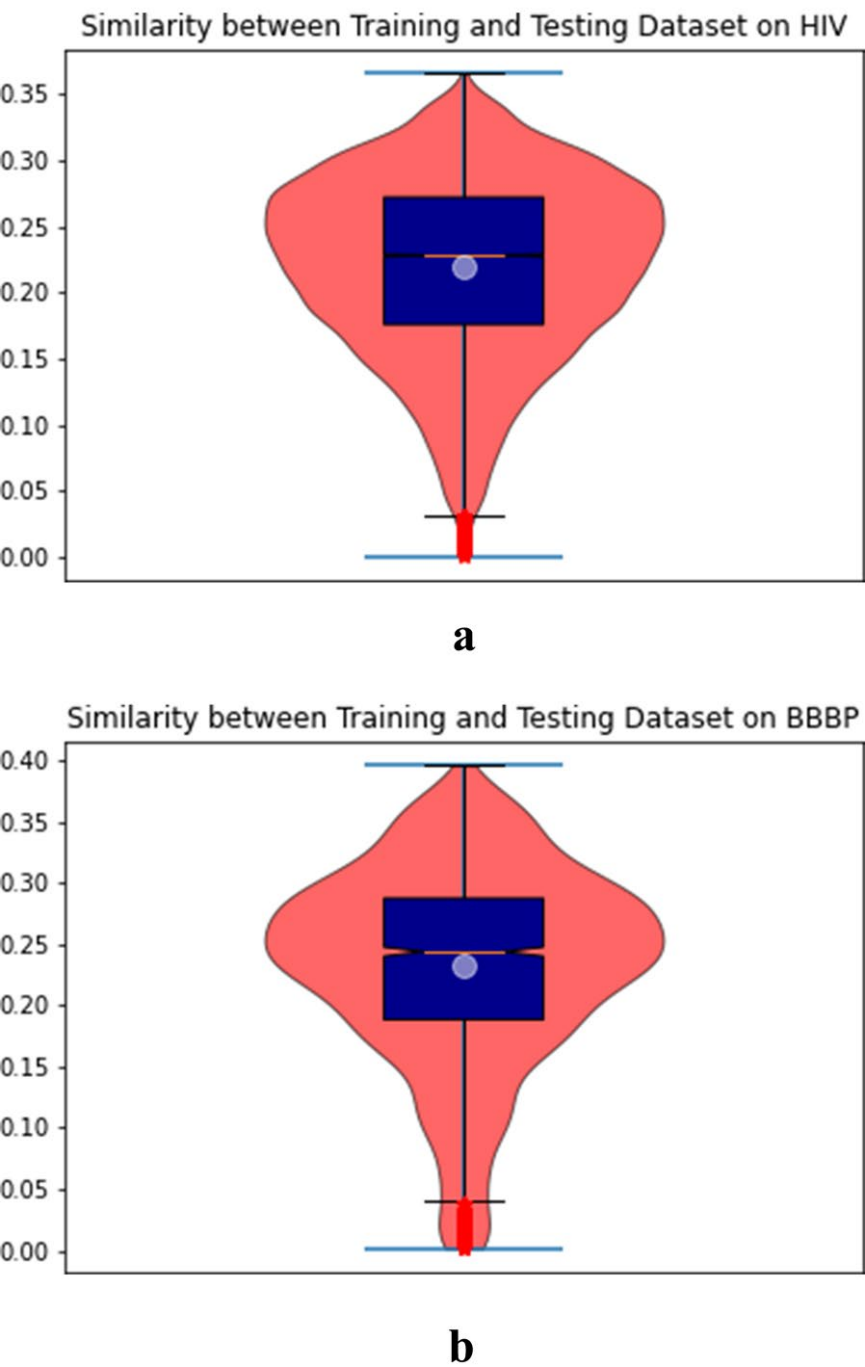
Similarity between training and testing datasets

The strong generalization ability of our FP-BERT based MPP model can be testified in light of the strict setting of the scaffold dataset splitting and the imbalance in datasets. The scaffold splitting ensures the dissimilarity between the training and testing datasets. We calculated the average similarity of each molecule on the training dataset to all the molecules on the testing dataset. The left violin plot of Fig. 6 shows the similarity between training and testing datasets on HIV and the right plot shows the similarity on BBBP.

The max similarity on HIV is 0.3652 and the median value is 0.2278 (as shown by a light blue ball), the mean value is 0.2199 (as shown by a red line). The max similarity on BBBP is 0.3956 and the median value is 0.2436, the mean value is 0.2317. The result certifies the molecules in the testing dataset are dissimilar to the molecules in the training dataset.

To explore the reason for low PR values on HIV, we computed the ratio of positive samples on classification datasets. The ratio of positive samples on HIV is only 0.035 compared to that of 0.765 on BBBP. Thus, the false positive predictions may exert a great influence on the model precision on HIV. The highly imbalance on the HIV dataset possibly causes the much lower PR values than that on BBBP.

### Experiments on regression tasks

For regression tasks, we evaluate our MPP models on the ESOL, FreeSolv Lipophilicity, Malaria and CEP datasets. To evaluate the performance of the prediction model in regression tasks, we report the average RMSE of five independent experiments on the test set and use the standard deviations to measure the stability of the model. We also provide $R^2$, $Q^2$ and the p-value from t test in terms of RMSE values of FP-BERT versus FP2Vec to further certify the regression performance of FP-BERT. The results are shown in Tables 2, 3 and 4. The benchmark results are directly taken from the literature [7, 9, 25] and [26].

In Tables 2 and 3, similar to the classification tasks, our models and the MolBERT still show better regression performance. The Ours-2 model provides the best performance on FreeSolv, the Ours-1 achieves the lowest RMSE value on CEP. Meanwhile, the Ours-1 and Ours-2 models achieve close performance. The MolBERT, possibly due to its sophisticated pre-training strategies, captures the best results on ESOL and Lipophilicity. Our models still achieve comparative results, and capture superior RMSE metrics to that of the FP2VEC and FCNN on ESOL and Lipophilicity. For the Malaria dataset, our FP-BERT based models achieve a slightly weaker performance than that of FP2VEC but are still superior to the other benchmark models.

The above observations can indicate the high performance of our MPP models for regression tasks. And our proposed models perform generally better than other molecular fingerprint-based methods, those are the FP2VEC, FCNN nad ECFP, in regression tasks. Furthermore, our FP-BERT featurizer can effectively learn molecular representation using 2 million compounds, but the featurizer promotes a little with the bigger dataset.

**Table 2:** The RMSE scores on test sets for the ESOL, FreeSolv and Lipophilicity datasets

Model	Featurizer	ESOL	FreeSolv	Lipophilicity
Ours-1	FP-BERT (2M)	0.67 ± 0.04	1.14 ± 0.06	0.66 ± 0.02
Ours-2	FP-BERT (10M)	0.67 ± 0.07	**1.07** ± **0.18**	0.67 ± 0.02
FP2VEC	FP2VEC	1.06 ± 0.10	1.56 ± 0.22	0.84 ± 0.02
FCNN	ECFP	1.12 ± 0.15	1.87 ± 0.07	0.86 ± 0.01
MolBERT	MolBERT	**0.552** ± **0.07**	1.523 ± 0.66	**0.602** ± **0.01**

**Table 3:** The RMSE scores on test sets for the Malaria and CEP datasets

Featurizer	Network	Malaria	CEP
Ours-1	FP-BERT (2M)	1.03 ± 0.06	**1.21** ± **0.07**
Ours-2	FP-BERT (10M)	1.05 ± 0.02	1.22 ± 0.04
FP2VEC	CNN	**1.01** ± **0.02**	1.34 ± 0.04
ECFP	Linear	1.13 ± 0.03	2.63 ± 0.09
	Neural network	1.36 ± 0.10	2.00 ± 0.09

**Fig. 7:**
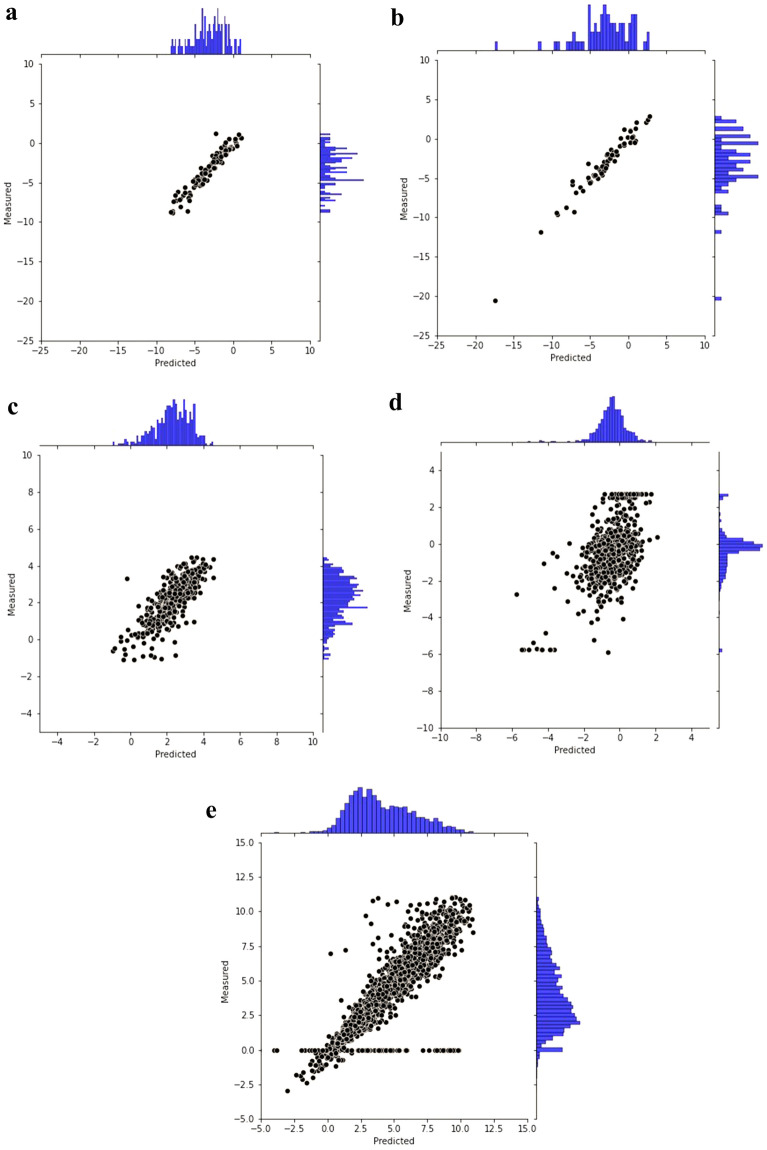
RMSE values of our MPP model versus measured values

Figure 7 illustrates the RMSE values of our MPP model versus observed properties on the five regression datasets, wherein the model is pre-trained on 10 million data. A perfect model is expected to provide a $y_i = t_i$ line where $y_i$ means the predicted value and $t_i$ means the ground truth. We observed that the densities of scatter points in the five sub figures were high around the $y_i = t_i$ line. That indicates our model can capture accurate predictive results visually.

**Table 4:** The statistical regression metrics of FP-BERT

Datasets	$R^2$	$Q^2$ 6	*p*-value versus *RMSE* of FP2Vec
ESOL	0.998	0.889	0.001
FreeSolv	0.990	0.929	0.001
Lipophilicity	0.955	0.679	4.99E-05
Malaria	0.787	0.229	0.092
CEP	0.913	0.766	0.012

We also report the statistical metrics of our model to further evaluate its performance in regression tasks. We conducted the proposed method and the benchmarking FP2VEC method for five times independently, then the p-value was computed by paired t-test in terms of the RMSE values.

Our MPP model achieved impressive $R^2$ and $Q^2$ values on 4 out of 5 datasets, demonstrating the FP-BERT model has high accuracies of fitness and prediction on the 4 regression tasks. We can also testify that our MPP model outperforms FP2VEC with p-values of far lower than 0.05 on the 4 out of 5 regression tasks. However, FP-BERT captures p-value = 0.092 on the Malaria dataset, demonstrating that the RMSE difference between our model and FP2VEC is not significant statistically on that dataset.

### Conclusions

This paper proposed a molecular property prediction method FP-BERT based on the pre-trained language model BERT and the CNN based prediction model. The pre-trained BERT model treats each substructure of a compound as a word and treats each compound as a sentence and it is used to encode each molecule. And the CNN block is used to extract high-level features from the learned molecular representation, then the fully connected layer is used to predict and output the property prediction result of each molecule. Experimental results showed that the proposed method achieves good performance in both regression and classification tasks, demonstrating the strong molecular representation ability of FP-BERT and proving that it is feasible to apply the concepts and techniques in NLP to computational biology. However, there are still some limitations of this study, the model can only predict the same properties as the dataset it was trained on, and this may be improved by the multi-task learning in the future.

## Data Availability

The source code is available on GitHub (https://github.com/fanganpai/fp2bert). We used the E15 dataset that is the diverse real drug-like 15.5M-molecule subset of the ENA dataset provided by the Enamine Corporation. The dataset we used can be downloaded from https://figshare.com/articles/dataset/Compound_dataset_for_pre-training/19092248 or the public 10.6084/m9.figshare.19092248. The whole ENA dataset can be found on https://2019-ncovgroup.github.io/data/#dataset-downloads. The five datasets for downstream regression tasks can be checked and downloaded on the website: https://figshare.com/articles/dataset/Untitled_Item/19091303 or the public 10.6084/m9.figshare.19091303, and the HIV and BBBP datasets for downstream classification tasks can be checked and downloaded on the website: https://figshare.com/articles/dataset/Dataset_for_classification/19091264 or the public 10.6084/m9.figshare.19091264. The outputted intermediate result of learned molecular embedding is shared on the website: https://figshare.com/articles/software/fingerprints_smile_output256_tar_gz/19609440 or the public 10.6084/m9.figshare.19609440. The fine-tuned BERT models according to specific downstream datasets are available on the https://figshare.com/articles/dataset/FP2BERT_embedding/19573084 or the public 10.6084/m9.figshare.19573084.
